# Delayed polarization of mononuclear phagocyte transcriptional program by type I interferon isoforms

**DOI:** 10.1186/1479-5876-3-24

**Published:** 2005-06-13

**Authors:** David F Stroncek, Christopher Basil, Dirk Nagorsen, Sara Deola, Eleonora Aricó, Kina Smith, Ena Wang, Francesco M Marincola, Monica C Panelli

**Affiliations:** 1 Department of Transfusion Medicine, Warren G. Magnuson Clinical Center, National Institutes of Health, Bethesda, Maryland, USA; 2 Charite – Universitatsmedizin Berlin, Campus Benjamin Franklin, Medizinische Klinik III, Hamatologie, Onkologie und Transfusionmedizin, Hindenburgdamm 30, Berlin, Germany

**Keywords:** Interferon, macrophages, autoimmunity

## Abstract

**Background:**

Interferon (IFN)-α is considered a key modulator of immunopathological processes through a signature-specific activation of mononuclear phagocytes (MPs). This study utilized global transcript analysis to characterize the effects of the entire type I IFN family in comparison to a broad panel of other cytokines on MP previously exposed to Lipopolysaccharide (LPS) stimulation in vitro.

**Results:**

Immature peripheral blood CD14+ MPs were stimulated with LPS and 1 hour later with 42 separate soluble factors including cytokines, chemokines, interleukins, growth factors and IFNs. Gene expression profiling of MPs was analyzed 4 and 9 hours after cytokine stimulation. Four hours after stimulation, the transcriptional analysis of MPs revealed two main classes of cytokines: one associated with the alternative and the other with the classical pathway of MP activation without a clear polarization of type I IFNs effects. In contrast, after 9 hours of stimulation most type I IFN isoforms induced a characteristic and unique transcriptional pattern separate from other cytokines. These "signature" IFNs included; IFN-β, IFN-α2b/α2, IFN-αI, IFN-α2, IFN-αC, IFN-αJ1, IFN-αH2, and INF-α4B and induced the over-expression of 44 genes, all of which had known functional relationships with IFN such as myxovirus resistance (Mx)-1, Mx-2, and interferon-induced hepatitis C-associated microtubular aggregation protein. A second group of type I IFNs segregated separately and in closer association with the type II IFN-γ. The phylogenetic relationship of amino acid sequences among type I IFNs did not explain their sub-classification, although differences at positions 94 through 109 and 175 through 189 were present between the signature and other IFNs.

**Conclusion:**

Seven IFN-α isoforms and IFN-β participate in the late phase polarization of MPs conditioned by LPS. This information broadens the previous view of the central role played by IFN-α in autoimmunity and tumor rejection by including and/or excluding an array of related factors likely to be heterogeneously expressed by distinct sub-populations of individuals in sickness or in response to biological therapy.

## Background

It has been proposed recently that autoimmunity or, more generally, immunity is driven by a dynamic system balancing opposite vectors represented by type one interferons (IFNs) in one direction and tumor necrosis factor (TNF) in the other [1]. This dichotomy is mediated through divergent activation of mononuclear phagocytes (MPs) by the two cytokine families resulting in specific signatures of gene expression that are characteristic of distinct autoimmune pathologies such as systemic lupus erythematosus (SLE, IFN-α/β) or rheumatoid arthritis (TNF) [1-3].

Interestingly, although type I IFN signatures are consistently observed in patients with SLE, only a fraction of them demonstrate elevated IFN serum protein levels [2]. Therefore, it is possible that testing methods may not detect all the IFN species that might circulate in the blood and which may yield similar biological effects. For this reason, we evaluated the effect of a panel of related type I IFN molecules in modulating the transcriptional profile of MPs exposed to lipopolysacaride (LPS) according to a previously described experimental model [4]. The molecular signatures of type I IFNs were compared with those of IFN-γ, TNF-α and TNF-β as well as a comprehensive panel of other cytokines to characterize the specificity of IFN polarization of MPs in the context of an extended cytokine network.

Indeed, MPs play a critical role in acute and chronic inflammation performing diverse functions in different stages of activation. MPs recruit and activate immune effector cells or they may down-regulate the inflammatory process to contain collateral damage [5]. Pro-inflammatory MPs, known as type 1 or M1 MPs, function as true antigen presenting cells that recruit and activate immune effector cells [6,7]. MP that enhance tissue repair, stimulate angiogenesis, and contain collateral damage through reduced inflammation are known as type 2, or M2 MPs [8,9]. Type 2 mononuclear phagocytes can be further categorized as M2 a, b, c [10] suggesting a continuous spectrum of MP activation.

Many factors may influence the differentiation of immature MPs toward a polarized M1 or M2 phenotype along the classical or the alternative pathway of transcriptional activation [9]. Theoretically, the *in vivo *functional phenotype of MPs along each of these pathways is dependent on the environment, since foreign products (microbial products) and endogenous cytokines modulate their activation and maturation [11]. MPs are capable of tailoring their response to specific microbes and microbial products as demonstrated by the display of a distinct gene expression profile upon stimulation with different pathogen products [11]. The effects of different cytokines and other bioactive soluble factors on MPs are also variable. Stimulation of MPs with interferon-γ (IFN-γ) alone or with lipopolysacaride (LPS) plus IFN-γ, CD40L, and Flt-3 ligand (FLT3-L) yields M1 MPs. Treatment of MPs with IL-4 and IL-13 induces a M2 MP phentotype.

Changes in MPs following microbial stimulation do not occur as a single event over a brief period of time but in waves each lasting several hours. A comparison of MPs stimulated with 3 different microbes, *E. coli*, *C. albicans *and influenza found that gene expression was most rapidly induced by *E. coli *and most slowly by influenza. The gene expression profiles induced by these three microbes continued to change for more than 24 hours [11].

We have found that cytokines have a marked affect on LPS-stimulated MPs [7]. Immature CD14+ peripheral blood MPs were stimulated with LPS and one hour later they were treated separately with 42 different cytokines. After 4 hours, the MPs were analyzed by gene expression profiling using a 17 K cDNA chip. LPS-stimulation alone induced a gene expression profile typical of the classical pathway of MP activation, but which was markedly altered by additional cytokine stimulation. Hierarchical clustering of gene expression profiles of LPS-stimulated MPs treated with 42 factors for four hours revealed two main groups of cytokines. One group included IL-4, IL-13, TGF-α, TGF-β, and VEGF and represented the alternative pathway of MP activation. A second group included IFN-β, IFN-γ, CD40L, and FLT-3L generally associated with the classical pathway. Some cytokines such as IL-10, IL-1β, IL-15, IFN-αA and IFN-α2b did not separate into either of the two classes [4].

Interestingly, 4 hours after cytokine exposure, type I IFNs did not display similarities in MP transcriptional activation among themselves. Instead, the various IFN family members were scattered among the different cytokine sub classifications. This finding suggested that IFN polarization of MP activation following LPS exposure is a delayed event. In this study, therefore, we present the gene profiling of LPS-conditioned MPs 9 hours after exposure to 42 different cytokines as previously reported for the 4 hour time point [4]. Prolonged exposure (9 hours) to these bioactive factors revealed a unique polarization of MP maturation induced by most (but not all) members of the type I IFN family and singled out this group of factors for its ability to induce a distinctive MP gene expression profile.

## Materials and methods

### MP separation and FACS staining

Peripheral blood mononuclear cells (PBMC) from an HLA-A*0201 positive healthy Caucasian male donor age 35 were collected by lymph apheresis at the Department of Transfusion Medicine, NIH. PBMC were isolated by Ficoll gradient separation and frozen until analysis. After thawing, PBMC were kept overnight in 175 cm^2 ^tissue culture flasks (Costar, Cambridge, MA) in complete medium (CM) consisting of Iscove's medium (Biofluids, Rockville, MD) supplemented with 10% heat-inactivated human AB-serum (Gemini Bioproducts, Inc, Calabasas, CA), 10 mM hepes buffer (Cellgro, Mediatech, Inc. Herndon, VA), 0.03% L-glutamine (Biofluids), 100 U/ml Penicillin/Streptomycin (Biofluids), 10 μg/ml Ciprofloxacin (Bayer, West Haven, CT), and 0.5 mg/ml amphotericin B (Biofluids). Adherent and non-adherent cells were gently removed from the flask and centrifugated. MP were separated by negative selection using the MP isolation kit and an autoMACS system (both Miltenyi, Bergisch Gladbach, Germany). Before and after separation cells were stained with anti-CD14-FITC (Becton Dickinson, San Diego, CA), and analyzed using a FACScalibur flow cytometer and CellQuest software (Becton Dickinson).

### Stimulation of MP, RNA isolation

Negatively selected CD14+ cells were washed twice with serum free OPTI-MEM (OM) medium (GIBCO BRL) prepared similarly to CM. The final cell population which contained > 90% CD14+ cells was then seeded at the concentration of 1 × 10^6 ^/ ml in 10 ml OM in 25 cm^2 ^flasks (Falcon, Franklin Lakes, NJ) and stimulated with 5 μg/ml LPS (SIGMA St. Louis, MO) for one hour. No LPS was added to the non-stimulation control flask. After one hour, 42 cytokines, chemokines and soluble factors were added individually to the MP suspensions (IL-1α, IFN-αC, IFN-αJ1, IL-13, IFN-α4B, MIP-1β, IFN-αG, IL-12, IL-5, IL-4, VEGF, TGFβ, IL-8, IFN-α1, MIP-4 (Parc), Rantes, IFN-αWA, TGFα, IFN-γ, IFN-α2, BCA-1, IFN-αF2, IL-2, Flt-3 Ligand, MIP 1α, IFN-αK, IL-6, IFN-β, IFN-αH2, IFN-αβ2, CD40L, IL-3, Tarc, TNF-α, IFN-α2b, IL-10, Aldara, IL-1IL-15, IFN-A, TNF-GM-CSF) [4]. Nine hours after LPS stimulation, MP were harvested, washed twice in PBS, and lysed for RNA isolation using 700 μl RNeasy lysis buffer per 25 cm^2 ^flask, according to the manufacturer's protocol.

### Probe preparation, amplification, and hybridization to microarrays

Total RNA was isolated using RNeasy minikits (Qiagen). Amplified antisense RNA (aRNA) was prepared from total RNA (0.5–3 μg) according the protocol previously described by us [12,13]. Test samples were labeled with Cy5-dUTP (Amersham, Piscataway, NJ) while the reference sample (pooled normal donor PBMC) was labeled with Cy3-dUTP. Test-reference sample pairs were mixed and co-hybridized to 17 K-cDNA microarrays.

### Microarrays and statistical analyses

Hybridized arrays were scanned at 10-μm resolution on a GenePix 4000 scanner (Axon Instruments, Union City, CA) at variable PMT voltage to obtain maximal signal intensities with < 1% probe saturation. Resulting tiff images were analyzed via ArraySuite software (National Human Genome Research Institute, Bethesda, MD). Data were further analyzed using Cluster and Tree View software [14] and Partek Pro software (Partek Inc., St Charles, MO). The global gene expression profiling of 9 hour treated and untreated MP consisted of 49 experimental samples. Subsequent low stringency filtering (80% gene presence across all experiments and removal of genes that did not have at least in 1 of the samples a Log_2 _= 2; 4 ratio) selected 2,063 genes for further analysis. Clustering of experimental samples according to Eisen et al. was based on these genes. Gene ratios were average corrected across experimental samples and displayed according to the central method for display using a normalization factor as recommended by Ross [15].

## Results

### Genes differentially expressed 9 hours after LPS and Cytokine Stimulation

We have previously shown that the transcriptional profile of MPs stimulated by LPS and subjected for 4 hours to distinct cytokine stimulation can be polarized into two major subgroups and an third intermediate subgroup according to the type of cytokine used in culture [4]. IFN-γ stimulation induced transcriptional changes that are consistent with its pro-inflammatory activity and with the type I pathway of MP activation [10]. After 4 hours type I IFNs demonstrated a variety of individual transcriptional profiles and no type I IFN-specific signatures could be identified. On the contrary, in the present analysis, we observed that 9 hours after stimulation a number of type I IFNs congregated within a single cluster that was characterized by molecular signatures unique to these cytokines and was responsible for a unique MP polarization response. This pattern of IFN-induced polarization was identified as follows: unsupervised hierarchical clustering analysis was applied to the global data set of MPs treated separately with 42 cytokines for 9 hours after LPS stimulation. The data set was filtered to sort genes that were expressed in at least 80% of samples and whose expression was increased 3 or more fold in at least one sample. Filtering yielded 2,063 genes that would be most informative among the 17 K gene pool.

**Figure 1:**
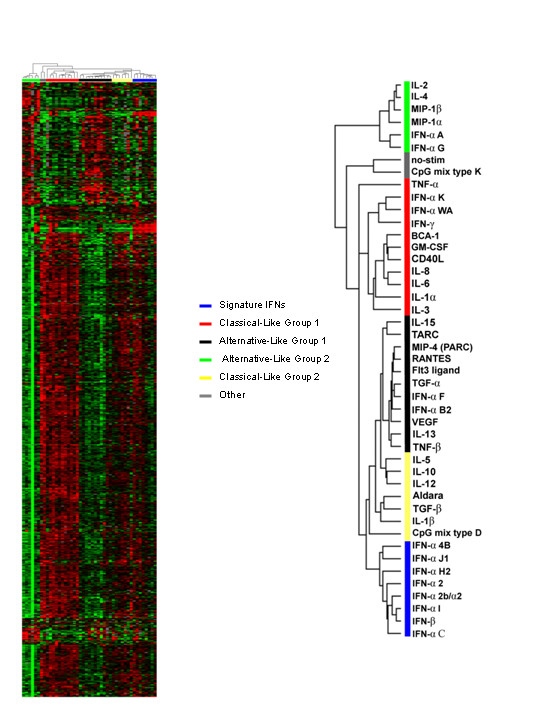
**Unsupervised clustering of LPS-stimulated CD14+ MP exposed to distinct cytokine treatments**. CD14+ MP were stimulated in parallel with LPS and exposed after 1 hour to 42 individual cytokines. Antisense RNA obtained 9 hours following LPS stimulation, was hybridized to custom made 17 k cDNA arrays. Unsupervised Eisen [20] clustering was applied to the complete, unfiltered data set of 98 experiments. A375 is a melanoma cell line that was used for quality control alternating conventional (Cy5, red) or reciprocal (Cy3, green) labeling every 25 experiments as previously described [18]. A total of 2063 genes were analyzed. These genes were obtained by filtering the 16,000 genes and analyzing only those present in greater than 80% of samples and with at least one observation greater than 2 logs. The expression of each gene was average corrected among the cytokines.

Cytokine treatments segregated into five groups (Figure 1). One group included cytokines best classified as those that act through the classical pathway of MP activation such as IFN-γ, TNF-α, IL-3, IL-6, GM-CSF, and CD40L (**Red bar**, Figure 1). These cytokines were similarly classified in our previous analysis at the 4 hour time point [4]. Since most though not all the cytokines in this group are recognized to act through the classical pathway of MP activation, this group was referred to as the "classical-like" group. Interestingly, this group included not only the type II IFN-γ but other type I IFNs such as IFN-αK and IFN-αWA. This observation suggested that these type I IFNs have similarities in the transcriptional activation of MPs with type II IFNs. Another cluster included cytokines that are characteristically included in the alternative pathway of MP activation such as IL-13, VEGF and TGF-α and it was referred to as the "alternative-like" group 1 (**Black bar**, Figure 1). On visual inspection this group displays a transcriptional profile opposite to the classical group. This group also included type I IFNs such as IFN-αB_2 _and IFN-αF or IFN-α21). A third group contained several cytokines that also activate MPs via the alternative pathway including IL-4, MIP-1α, and MIP-1β and was referred to as alternative-like group 2(**Green bar**, Figure 1). This group was most distant in transcriptional profile from all other groups and was separated from them by the transcriptional profile of MPs stimulated with oligonucleotides containing a unmethylated deoxycytosine-deoxyguanosine (CpG) motifs mixture (CpG mix type K) (**Grey bar**, Figure 1) or had not been exposed to any soluble factors and, therefore, was representative of the transcriptional profile of MPs in culture. Thus, this group most likely represented the most divergent example of alternative MP activation 9 hours after stimulation. However, even on visual inspection, the alternative-like group 2 displays a transcriptional pattern similar to the alternative-like group 1 with most genes co-coordinately expressed between them (Figure 1).

A fourth cluster included a variety of cytokines whose effect on MPs could not be precisely categorized. However, on visual inspection this group approximated the classical-like group. This group comprised IL-5, IL-10, IL-12 and TGF-β and was called "classical-like group 2"The fifth and most relevant to this study was a cluster we called "signature IFNs", that included only type I IFNs such as: IFN-β, IFN-α2/α2, IFN-αI, IFN-α2, IFN-αC, IFN-αJ1, IFN-αH2, and IFN-α4B. This grouping of type I IFN strongly suggested an IFN-specific pathway of MP polarization in the late phase of their transcriptional response to LPS.

Overall, 15 IFNs were tested, 13 IFN-α's, IFN-β and IFN-γ. The IFNs not included among the eight present in the fifth cluster included IFN-γ and IFN-αA, IFN-αG, IFN-αK, IFN-αWA, IFN-αF, IFN-αB2. IFN-αF and IFN-αB2 clustered with the Alternative-like group 1 group, while IFN-αA and IFN-αG with the Alternative-like group 2. IFN-αK, IFN-αWA, and IFN-γ clustered with the classical cytokines. Interestingly, the global transcript profile of the 8 type I signature IFNs was opposite for a large number of genes to that of classical-like cytokines which included TNF-α suggesting that, indeed, type I IFNs represent a distinct biological vector of MP maturation.

**Figure 2:**
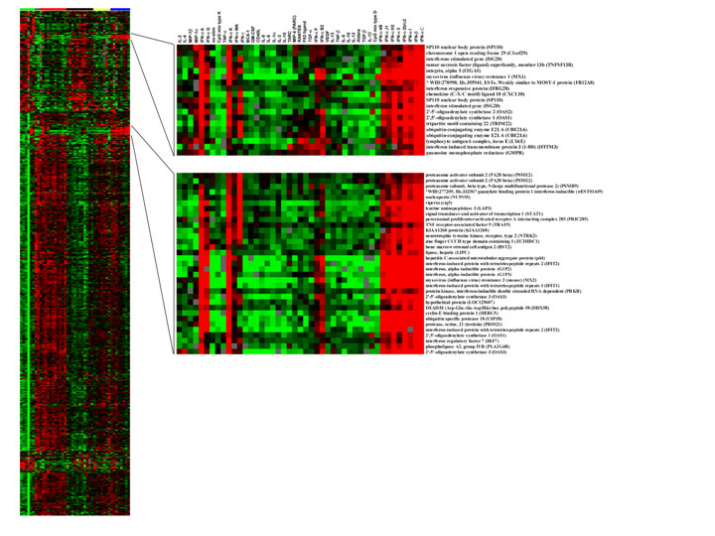
**The genes overexpressed by LPS-stimulated MPs treated by 8 type I IFNs**. Unsupervised hierarchical clustering was applied to LPS-stimulated CD14+ MP treated for 9 hours with 42 individual cytokines. FN-β and seven αFNs were clustered into a separate group, the signature IFNs. These IFNs were characterized by the marked overexpression of two groups of genes shown. The genes were overexpressed by the eight IFNs and were identified by visual inspection. The analysis was preformed on the same 2063 genes used in figure 1.

Two functional signatures appeared to be particularly restricted to signature IFNs. These two signatures included 52 cDNA clones representing 51 genes. In one signature 19 clones represented 18 genes and in the other and 33 clones represented 33 genes. Forty four of the 51 signature genes were unique. Duplicate copies of 7 genes were among 51 genes. These 44 genes characterized the unique transcriptional profile of type I IFN polarized MPs (Figure 2). All 44 signature genes were previously known to be associated with IFN function [16-49] (Table 1). Among them were included myxovirus resistance (Mx)-1, Mx-2, interferon-induced hepatitis C-associated microtubular aggregation protein, 2',5'-oligoadenylate synthetase 1 (OAS1), OAS2, and OAS3, vipirin (CIG5), IFN regulatory factor 7 (IRF-7), and lymphocyte antigen 6 complex (LY6E), Interferon stimulated gene (ISG20), Interferon responsive protein (IFRG28), Interferon induced transmembrane protein 3 (I-8D) (IFITM3), Interferon induced protein with tetratricopeptide 1 (IFIT1), Interferon induced protein with tetratricopeptide 2 (IFIT2), Interferon-alpha inducible protein (GIP2), Interferon-alpha inducible protein (GIP3), Protein kinase, interferon-inducible double stranded RNA activated protein kinase (PRKR), and Signal transducer and activator of transcription 1 (STAT1). Seven of these genes were duplicated in the array, nuclear body protein (SP110), OAS1, OAS3, ISG20, ubiquitin conjugated enzyme E2L6 (UBE2LB), PA28 proteasome subunit (PSME2), and IFIT2, and both copies of the seven genes were among the 52 cDNA clones. It is important to note that the non-signature IFNs demonstrated nearly identical expression of these genes while none of the other cytokine studied expressed them; underlining the selectivity of this expression pattern to type I IFNs with some overlap with IFN-γ.

**Table 1:** 44 Signature Genes that are Interferon Related

**Gene**	**Function/Association**	**Alias**	**Induced by most IFN-α's**	**Induced by IFN-β**	**Ref**
SP110	nuclear body protein	Interferon-induced protein 41/75	yes	yes	16
C1Orf29	Unknown		yes	yes	none
ISG20	IFN stimulated genes	HEM45	yes	yes	17–18
TNFSF13B	TFN superfamily	BAFF BAFF BLYS HGNC:13472 TALL-1 THANK TNFSF20 ZTNF4 (L	yes	yes	19
ITGA5	Integrin-alpha 5 (receptor for fibrinogen and fibronectin)	CD49e, FNRA, VLA5A, fibronectin receptor (alpha subunit), integrin alpha 5	yes	yes	20
Mx1	Mixovirus resistence		yes	no	21
IFRG28	IFN responsive, potential type III membrane protein		yes	no	none
CXCL10	CXC family	C7, IFI10, INP10, IP-10, SCYB10, crg-2, gIP-10, mob-1, chemokine (C-X-C motif) ligand 10, gamma IP10	yes	yes	22,23
OAS2	2'5'OAS 2		yes	yes	24
OAS1	2'5'OAS 1		yes	yes	25
TRIM22	STAF 50	GPSTAF50, RNF94, STAF50-PEN, Stimulated Trans-Acting Factor	yes	yes	25,26
UBE2L6	Ubiquitin conjugated enzyme	MGC40331, RIG-B, UBCH8, retinoic acid induced gene B protein, ubiquitin carrier protein, ubiquitin-conjugating enzyme E2L 6, ubiquitin-protein ligase, Ubiquitin-conjugating enzyme	yes	yes	27
Ly6E	Lymphocyte antigen complex	LY6, RIG-E, RIGE, SCA-2, SCA2, TSA-1	yes	yes	28
IFITM3	IFN-induced transmembran protein	1-8U	yes	no	29,30
GMPR	Guanosine monophosp reductase	Catalyzes the irreversible NADPH-dependent deamination of GMP to IMP	yes	no	none
PSME2	PA28 Proteasome subunit	PA28B, PA28beta, REGbeta, 11S regulator complex beta subunit, MCP activator	yes	yes	31, 32
PSMB9	Proteasome subunit	LMP2, RING12	yes	yes	32
GBP1	Binds GTP, GDP and GMP	Interferon-induced guanylate-binding protein 1	yes	yes	33
NUP155	nucleoporin	KIAA0791, N155	yes	yes	none
CIG5	antiviral protein induced by CMV	vig1, Viperin	yes	yes	34
LAP3	Signal transducer	HGNC:8843, LAP, LAPEP, PEPS	yes	yes	35
Stat1	Transcription factor that binds to the ISRE and GAS elements	ISGF-3, STAT91	yes	yes	36
PRIC 285	helicase	FLJ00244, KIAA1769	yes	yes	none
TRAF5	adapter protein and signal transducer	MGC:39780, RNF8	yes	yes	none
KIAA1268	unknown		yes	yes	none
NTRK2	tyrosine kinases receptor	TRKB	yes	yes	37
ZC3HDC1	zinc finger CCCH-type domain		yes	yes	none
BST2	bone marrow stromal antigen		yes	yes	none
LIPC	lipase, hepatic	HL, HTGL, LIPH, lipase hepatic	yes	no	none
IFI44	interferon-induced, hepatitis C-associated microtubular aggregate protein	MTAP44	yes	yes	38
IFIT2	IFN-α inducible protein	G10P2, GARG-39, IFI-54, IFI54, ISG-54K, cig42, Interferon alpha-inducible protein	yes	yes	39, 40
G1P2	IFN-α inducible protein, Ubiquitin cross-reactive protein precursor	IFI15, ISG15, UCRP	yes	yes	27
G1P3	IFN-α inducible protein	6–16, IFI616, interferon, alpha-inducible protein	yes	no	41
Mx2	myxo resistance		yes	no	21
IFIT1	IFN-α inducible protein	G10P1, GARG-16, IFI-56, IFI56, IFNAI1, RNM561	yes	no	42
PRKR	Interferon-induced, double-stranded RNA-activated protein kinase	EIF2AK1, PKR, interferon-inducible elF2alpha kinase	yes	yes	43
OAS3	2'5'OAS 3	p100, 2'-5'-oligoadenylate synthetase 3	yes	yes	24
LOC129607	hypotetical protein		yes	yes	none
Cyclin E binding protein 1	ubiquitin-protein ligase, regulation of cyclin dependent protein kinase activity	CEBP1, HECT E3 ubiquitin ligase	yes	no	44
DXD58	Retinoic acid-inducible gene	RIG-I, DEAD (Asp-Glu-Ala-Asp) box polypeptide 58	yes	no	45
USP18	ubiquitin specific protease 18	ISG43, UBP43, ubiquitin specific protease 18	yes	no	46, 47
PRSS21	protease serine, Could regulate proteolytic events associated with testicular germ cell maturation.	ESP-1, TEST1, testisin	yes	no	none
IRF7	IFN regulatory factor 7		yes	yes	48
PLA2G4B	phospholipase A2	CPLA2-BETA, HsT16992, PLA2G4B protein	yes	yes	49

* Interferon γ only

### Specificity of type I IFN signatures

**Figure 3:**
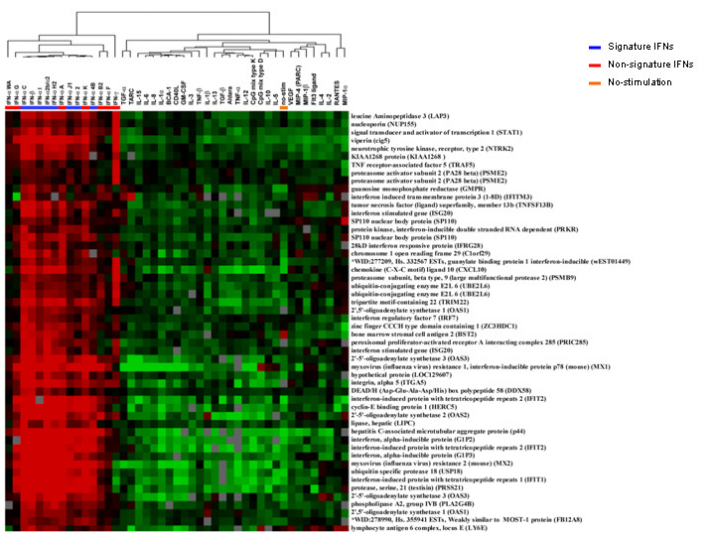
**Clustering of all 15 IFNs among the 54 genes overexpressed by LPS-stimulated MP treated with the eight signature IFNs**. Hierarchical clustering was applied to LPS-stimulated CD14+ MP treated for 9 hours with 42 individual cytokines. The analysis was limited to the 52 genes overexpressed by signature IFNs.

To determine whether the MP genes included in the IFN-specific signatures were specifically differentially expressed by all IFNs compared with other cytokines, we regrouped the various cytokine treatments according to hierarchical clustering analysis based on only the 44 signature genes (Figure 3). Indeed, the expression of all of the signature genes was shared by all type I IFNs and was partially shared by IFN-γ, which clustered in between type I IFNs and the other cytokines. The signature IFNs remained in a single group, but this group also included IFN-αA and IFN-αK. Thus, the differences in the transcriptional profile of MPs treated by distinct types of type I IFNs were not due to different activation of IFN-specific genes but rather by additional effects that distinct IFNs have on MP activation

**Figure 4:**
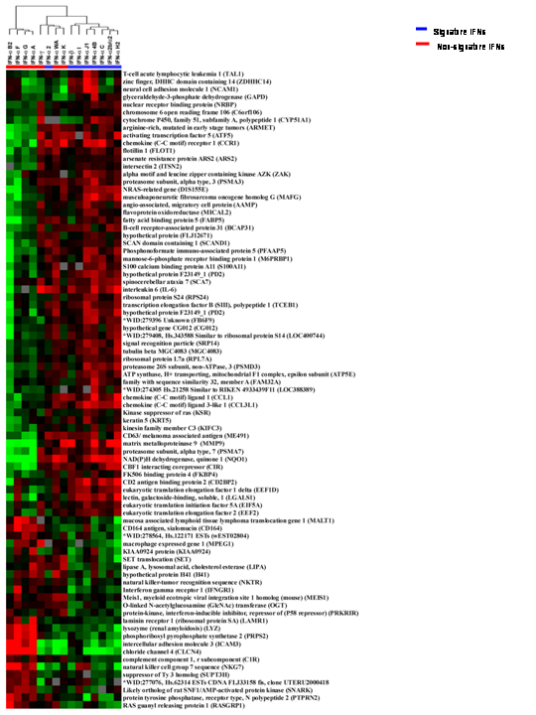
**Selection and clustering of genes differently expressed among LPS-stimulated MPs treated with the signature IFNs and non-signature IFNs**. Unsupervised hierarchical clustering was applied to LPS-stimulated CD14+ MP treated for 9 hours with the IFNs. The analysis was restricted to genes that were differentially expressed between the signature and non-signature INFs (p < 0.05), but the 52 genes markedly overexpressed by LPS-stimulated MPs treated with the signature IFNs were excluded from the analysis. The signature IFNs are shown in blue and the non-signature IFNs are shown in red.

To determine the genes responsible for the differentiation between signature and non-signature IFNs, we identified genes statistically differentially expressed between the two groups. The expression of 83 genes differed significantly between the signature and non-signature IFNs (unpaired *t *test p_2_-value < 0.05) (Figure 4). The signature IFNs remained in a single group, but this group also included IFN-αWA and IFN-αK. The expression of a specific set of genes in the node spanning from the CD164 antigen to protein-kinase, interferon-inducible repressor (PRKRIR) resulted in the grouping of IFN-αWA and IFN-αK with the signature IFNs.

By using a data base for annotation visualization and integrated discovery , genes differentially expressed between signature-and non-signature-IFNs were categorized according to specific cellular and molecular functions. Sixteen functional groups each containing at least 3 representative genes belonging to a particular biological process were selected: 1) Metabolism/protein metabolism; 2) Catalytic activity/ hydrolase activity; 3) Cell proliferation; 4) Cell growth and maintanance; 5) Cell cycle; 6) Regulation of transcription; 7) Nuclear binding; 8) Nucleobase/ nucleoside and nucleic acid metabolism; 9) Signal transducer activity; 10) Immune response/defence; 11) Cell adhesion; 12) Development; 13) biosynthesis/regulation of translation; 14) Cytoskeleton 15) Transport 16) Cell death (Table 2, also refer to this table for details on gene name abbreviations and single gene function).

**Table 2:** Functional grouping of genes differentially expressed between signature and non-signature IFNs

**GENENAME**	**GENE DEESCRIPTION**	**LOCUSLINK CLASSIFICATIONS**	**UNIQID**	**LOCUSLINK**
**1)Metabolism/protein metabolism**				
ZAK	sterile alpha motif and leucine zipper containing kinase AZK	ATP binding; DNA damage response, signal transduction resulting in cell cycle arrest; MAP kinase kinase kinase activity; activation of JUNK; activation of MAP/ERK kinase kinase; activation of MAPKK; cell cycle arrest; cell death; cell differentiation; cell proliferation; cellular_component unknown; protein amino acid phosphorylation; protein serine/threonine kinase activity; protein-tyrosine kinase activity; response to radiation; response to stress; transferase activity	Hs.115175	51776
MMP9	matrix metalloproteinase 9 (gelatinase B, 92 kDa gelatinase, 92 kDa type IV collagenase)	collagen catabolism; collagenase activity; extracellular matrix; extracellular space; gelatinase B activity; hydrolase activity; zinc ion binding	Hs.151738	4318
GAPD	glyceraldehyde-3-phosphate dehydrogenase	cytoplasm; glucose metabolism; glyceraldehyde-3-phosphate dehydrogenase (phosphorylating) activity; glycolysis; oxidoreductase activity	Hs.169476	2597
PRKRIR	protein-kinase, interferon-inducible double stranded RNA dependent inhibitor, repressor of (P58 repressor)	DNA binding; negative regulation of cell proliferation; protein binding; regulation of translation; response to stress; signal transduction	Hs.177574	5612
SRP14	signal recognition particle 14 kDa (homologous Alu RNA binding protein)	7S RNA binding; cotranslational membrane targeting; protein targeting; signal recognition particle	Hs.180394	6727
MALT1	mucosa associated lymphoid tissue lymphoma translocation gene 1	activation of NF-kappaB-inducing kinase; anti-apoptosis; caspase activity; defense response; intracellular; peptidase activity; protein binding; proteolysis and peptidolysis; signal transduction	Hs.180566	10892
PSMA7	proteasome (prosome, macropain) subunit, alpha type, 7	endopeptidase activity; proteasome core complex (sensu Eukarya); ubiquitin-dependent protein catabolism	Hs.233952	5688
PSMA3 *	proteasome (prosome, macropain) subunit, alpha type, 7	endopeptidase activity; proteasome core complex (sensu Eukarya); ubiquitin-dependent protein catabolism	Hs.346918	5684
LYZ	lysozyme (renal amyloidosis)	carbohydrate metabolism; cell wall catabolism; cytolysis; defense response to bacteria; extracellular space; hydrolase activity, acting on glycosyl bonds; inflammatory response; lysozyme activity	Hs.234734	4069
MAFG	v-maf musculoaponeurotic fibrosarcoma oncogene homolog G (avian)	chromatin; nucleus; regulation of transcription, DNA-dependent; transcription factor activity; transcription from Pol II promoter	Hs.252229	4097
NRBP	nuclear receptor binding protein	ATP binding; SH3/SH2 adaptor protein activity; nucleus; protein amino acid phosphorylation; protein kinase activity; receptor activity; signal transduction; transferase activity	Hs.272736	29959
SCAND1	SCAN domain containing 1	RNA binding; nucleus; protein self binding; regulation of cell cycle; regulation of cell proliferation; regulation of transcription, DNA-dependent; transcription factor activity; transcription factor binding	Hs.274411	51282
EEF1D	eukaryotic translation elongation factor 1 delta (guanine nucleotide exchange protein)	eukaryotic translation elongation factor 1 complex; guanyl-nucleotide exchange factor activity; protein biosynthesis; translation elongation factor activity; translational elongation	Hs.334798	1936
NQO1	NAD(P)H dehydrogenase, quinone 1	NAD(P)H dehydrogenase (quinone) activity; cytochrome-b5 reductase activity; cytoplasm; electron transport; nitric oxide biosynthesis; oxidoreductase activity; response to toxin; synaptic transmission, cholinergic; xenobiotic metabolism	Hs.406515	1728
FABP5	fatty acid binding protein 5 (psoriasis-associated)	cytoplasm; epidermal differentiation; fatty acid binding; lipid metabolism; transport; transporter activity	Hs.408061	2171
S100A11	S100 calcium binding protein A11 (calgizzarin)	calcium ion binding; cytoplasm; negative regulation of DNA replication; negative regulation of cell proliferation; nucleus	Hs.417004	6282
DIS155E	NRAS-related gene	DNA binding; RNA binding; male gonad development; regulation of transcription, DNA-dependent	Hs.69855	7812
TAL1	T-cell acute lymphocytic leukemia 1	DNA binding; cell differentiation; cell proliferation; regulation of transcription, DNA-dependent	Hs.73828	6886
PTPRN2	protein tyrosine phosphatase, receptor type, N polypeptide 2	hydrolase activity; integral to plasma membrane; protein amino acid dephosphorylation; protein-tyrosine-phosphatase activity; receptor activity; transmembrane receptor protein tyrosine phosphatase activity	Hs.74624	5799
FKBP4	FK506 binding protein 4, 59 kDa	FK506 binding; Hsp70/Hsp90 organizing protein activity; biological_process unknown; cytoplasm; isomerase activity; nucleus; peptidyl-prolyl cis-trans isomerase activity; protein folding	Hs.848	2288
LIPA	lipase A, lysosomal acid, cholesterol esterase (Wolman disease)	N-linked glycosylation; hydrolase activity; lipid catabolism; lipoprotein lipase activity; lysosome; sterol esterase activity	Hs.85226	3988
ATF5	activating transcription factor 5	DNA binding; RNA polymerase II transcription factor activity; nucleus; regulation of cell cycle; regulation of transcription from Pol II promoter; transcription corepressor activity	Hs.9754	22809
CD63*	lysosomal membrane glycoprotein	CD63 serves as an adaptor protein that links its interaction partners to the endocytic machinery of the cell/trafficking role	Hs.445570	29186
LAMR1	laminin receptor 1 (ribosomal protein SA, 67 kDa)	cell adhesion; cell surface receptor linked signal transduction; cytosolic small ribosomal subunit (sensu Eukarya); integrin complex; intracellular; laminin receptor activity; protein biosynthesis; regulation of translation; structural constituent of ribosome	Hs.181357	3921
**2) Catalytic activity/hydrolase activity**				
ZAK	sterile alpha motif and leucine zipper containing kinase AZK	(See description above)	Hs.115175	51776
MMP9	matrix metalloproteinase 9 (gelatinase B, 92 kDa gelatinase, 92 kDa type IV collagenase)	(See description above)	Hs.151738	4318
GAPD	glyceraldehyde-3-phosphate dehydrogenase	(See description above)	Hs.169476	2597
MALT1	mucosa associated lymphoid tissue lymphoma translocation gene 1	(See description above)	Hs.180566	10892
PSMA7	proteasome (prosome, macropain) subunit, alpha type, 7	(See description above)	Hs.233952	5688
PSMA3 *	proteasome (prosome, macropain) subunit, alpha type, 7	(See description above)	Hs.346918	5684
LYZ	lysozyme (renal amyloidosis)	(See description above)	Hs.234734	4069
NRBP	nuclear receptor binding protein	(See description above)	Hs.272736	29959
FLJ12671	hypothetical protein FLJ12671	exonuclease activity; hydrolase activity; nucleus	Hs.301904	81875
NQO1	NAD(P)H dehydrogenase, quinone 1	(See description above)	Hs.406515	1728
PTPRN2	protein tyrosine phosphatase, receptor type, N polypeptide 2	(See description above)	Hs.74624	5799
FKBP4	FK506 binding protein 4, 59 kDa	(See description above)	Hs.848	2288
LIPA	lipase A, lysosomal acid, cholesterol esterase (Wolman disease)	(See description above)	Hs.85226	3988
**3) Cell proliferation**				
ZAK	sterile alpha motif and leucine zipper containing kinase AZK	(See description above)	Hs.115175	51776
PRKRIR	protein-kinase, interferon-inducible double stranded RNA dependent inhibitor, repressor of (P58 repressor)	(See description above)	Hs.177574	5612
SCAND1	SCAN domain containing 1	(See description above)	Hs.274411	51282
H41	hypothetical protein H41	cell proliferation; cellular_component unknown; molecular_function unknown	Hs.283690	55573
S100A11	S100 calcium binding protein A11 (calgizzarin)	(See description above)	Hs.417004	6282
CD164	CD164 antigen, sialomucin	cell adhesion; development; endosome; hemopoiesis; immune response; integral to plasma membrane; membrane fraction; negative regulation of cell adhesion; negative regulation of cell proliferation; signal transduction; soluble fraction	Hs.43910	8763
TAL1	T-cell acute lymphocytic leukemia 1	(See description above)	Hs.73828	6886
ATF5	activating transcription factor 5	(See description above)	Hs.9754	22809
IL-6	Interleukin 6	reactants also involved in the regulation of immune response, hematopoiesis, platelet production, acute phase reaction and bone resorption (susceptibility factor for osteopenia), playing a role in the aggressiveness of non hodgkin lymphoma by stimulating MMP2 and MMP9	Hs.512234	3569
**4) Cell growth and maintanance**				
SCA7	spinocerebellar ataxia 7 (olivopontocerebellar atrophy with retinal degeneration)	molecular_function unknown; nuclear organization and biogenesis; nucleus; visual perception	Hs.108447	6314
ZAK	sterile alpha motif and leucine zipper containing kinase AZK	(See description above)	Hs.115175	51776
TIP47	mannose-6-phosphate receptor binding protein 1	Golgi apparatus; endosome; receptor activity; vesicle-mediated transport	Hs.140452	10226
PRKRIR	protein-kinase, interferon-inducible double stranded RNA dependent inhibitor, repressor of (P58 repressor)	(See description above)	Hs.177574	5612
SRP14	signal recognition particle 14 kDa (homologous Alu RNA binding protein)	(See description above)	Hs.180394	6727
TUBB5	tubulin beta MGC4083	GTP binding; microtubule; microtubule-based movement; structural molecule activity	Hs.274398	84617
SCAND1	SCAN domain containing 1	(See description above)	Hs.274411	51282
H41	hypothetical protein H41	(See description above)	Hs.283690	55573
FABP5	fatty acid binding protein 5 (psoriasis-associated)	(See description above)	Hs.408061	2171
S100A11	S100 calcium binding protein A11 (calgizzarin)	(See description above)	Hs.417004	6282
CD164	CD164 antigen, sialomucin	(See description above)	Hs.43910	8763
CCL1	chemokine (C-C motif) ligand 1	calcium ion homeostasis; cell-cell signaling; chemokine activity; chemotaxis; extracellular space; immune response; inflammatory response; signal transduction; viral life cycle	Hs.72918	6346
TAL1	T-cell acute lymphocytic leukemia 1	(See description above)	Hs.73828	6886
ATF5	activating transcription factor 5	(See description above)	Hs.9754	22809
**5) Cell cycle**				
ZAK	sterile alpha motif and leucine zipper containing kinase AZK	(See description above)	Hs.115175	51776
SCAND1	SCAN domain containing 1	(See description above)	Hs.274411	51282
S100A11	S100 calcium binding protein A11 (calgizzarin)	(See description above)	Hs.417004	6282
ATF5	activating transcription factor 5	(See description above)	Hs.9754	22809
**6) Regulation of transcription**				
MAFG	v-maf musculoaponeurotic fibrosarcoma oncogene homolog G (avian)	chromatin; nucleus; regulation of transcription, DNA-dependent; transcription factor activity; transcription from Pol II promoter	Hs.252229	4097
SCAND1	SCAN domain containing 1	(See description above)	Hs.274411	51282
D1S155E	NRAS-related gene	(See description above)	Hs.69855	7812
TAL1	T-cell acute lymphocytic leukemia 1	(See description above)	Hs.73828	6886
ATF5	activating transcription factor 5	(See description above)	Hs.9754	22809
**7) Nucleotide binding**				
ZAK	sterile alpha motif and leucine zipper containing kinase AZK	(See description above)	Hs.115175	51776
KIFC3	kinesin family member C3	ATP binding; microtubule associated complex; motor activity	Hs.23131	3801
NRBP	nuclear receptor binding protein	(See description above)	Hs.272736	29959
TUBB-5	tubulin beta MGC4083	(See description above)	Hs.274398	84617
**8)Nucleobase nucleoside and nucleic acid metabolism**				
MAFG	v-maf musculoaponeurotic fibrosarcoma oncogene homolog G (avian)	(See description above)	Hs.252229	4097
SCAND1	SCAN domain containing 1	(See description above)	Hs.274411	51282
S100A11	S100 calcium binding protein A11 (calgizzarin)	(See description above)	Hs.417004	6282
D1S155E	NRAS-related gene	(See description above)	Hs.69855	7812
TAL-1	T-cell acute lymphocytic leukemia 1	(See description above)	Hs.73828	6886
ATF-5	activating transcription factor 5	(See description above)	Hs.9754	22809
**9) Signal transducer activity**				
ZAK	sterile alpha motif and leucine zipper containing kinase AZK	(See description above)	Hs.115175	51776
TIP47	mannose-6-phosphate receptor binding protein 1	Golgi apparatus; endosome; receptor activity; vesicle-mediated transport	Hs.140452	10226
NRBP	nuclear receptor binding protein	(See description above)	Hs.272736	29959
CCR1	chemokine (C-C motif) receptor 1	C-C chemokine receptor activity; G-protein signaling, coupled to cyclic nucleotide second messenger; cell adhesion; cell-cell signaling; chemotaxis; cytosolic calcium ion concentration elevation; immune response; inflammatory response; integral to plasma membrane; rhodopsin-like receptor activity	Hs.301921	1230
CCL1	chemokine (C-C motif) ligand 1	calcium ion homeostasis; cell-cell signaling; chemokine activity; chemotaxis; extracellular space; immune response; inflammatory response; signal transduction; viral life cycle	Hs.72918	6346
PTPRN2	protein tyrosine phosphatase, receptor type, N polypeptide 2	hydrolase activity; integral to plasma membrane; protein amino acid dephosphorylation; protein-tyrosine-phosphatase activity; receptor activity; transmembrane receptor protein tyrosine phosphatase activity	Hs.74624	5799
**10) Immune response/ defence**				
CD2BP2	CD2 antigen (cytoplasmic tail) binding protein 2	antimicrobial humoral response (sensu Vertebrata); cytoplasm	Hs.202677	10421
LYZ	lysozyme (renal amyloidosis)	(See description above)	Hs.234734	4069
CCR1	chemokine (C-C motif) receptor 1	(See description above)	Hs.301921	1230
CD164	CD164 antigen, sialomucin	(See description above)	Hs.43910	8763
CCL1	chemokine (C-C motif) ligand 1	(See description above)	Hs.72918	6346
MALT1	mucosa associated lymphoid tissue lymphoma translocation gene 1	activation of NF-kappaB-inducing kinase; anti-apoptosis; caspase activity; defense response; intracellular; peptidase activity; protein binding; proteolysis and peptidolysis; signal transduction	Hs.180566	10892
NKG7*	Natural killer cell group 7 sequence	Expressed in activated T cells, alpha LAK cells in kidney, liver, lung and pancreas. Not expressed in brain, heart, or skeletal muscle. Expressed at high levels in TCR gamma delta-expressing CTL clones, and in some TCR alpha beta-expressing CTL clones (both CD4+ and CD8+), but is not expressed in other TCR alpha beta-expressing CTL clones and in cell lines representing Bcells, monocytes, and myeloid cells	Hs.10306	4818
NKTR*	NK cell tumor recognition protein	Activation of NK cell eleveted levels upon recognition of tumors by NK	Hs.432885	4820
MPEG1*	likely ortholog of mouse macrophage expressed gene 1	Mpg-1 is a novel gene that may share a distant ancestry to perforin, a lytic protein found in cytotoxic T lymphocytes and natural killer cells	Hs.549280	219972
CCL3L1*	chemokine (C-C motif) ligand 3-like 1	chemokine activity; chemotaxis; extracellular; immune response; inflammatory response; negative regulation of cell proliferation	Hs.387650	6349
IL-6	Interleukin 6	(See description above)	Hs.512234	3569
**11) Cell adhesion**				
CCR1	chemokine (C-C motif) receptor 1	(See description above)	Hs.301921	1230
CD164	CD164 antigen, sialomucin	(See description above)	Hs.43910	8763
FKBP4	uncharacterized hematopoietic stem/progenitor cells protein MDS028	cell-matrix adhesion; integrin complex	Hs.848	55846
LAMR1*	laminin receptor 1 (ribosomal protein SA, 67 kDa)	(See description above)	Hs.181357	3921
ICAM3*	intracellular adhesion molecule 3	cell-cell adhesion; integral to plasma membrane; integrin binding; protein binding	Hs.99995	3385
**12) Development**				
ZAK	sterile alpha motif and leucine zipper containing kinase AZK	(See description above)	Hs.115175	51776
FABP5	fatty acid binding protein 5 (psoriasis-associated)	cytoplasm; epidermal differentiation; fatty acid binding; lipid metabolism; transport; transporter activity	Hs.408061	2171
KRT5	keratin 5 (epidermolysis bullosa simplex, Dowling-Meara/Kobner/Weber-Cockayne types)	epidermal differentiation; intermediate filament; structural constituent of cytoskeleton	Hs.433845	3852
CD164	CD164 antigen, sialomucin	(See description above)	Hs.43910	8763
D1F155E	NRAS-related gene	(See description above)	Hs.69855	7812
TAL1	T-cell acute lymphocytic leukemia 1	(See description above)	Hs.73828	6886
**13) Biosynthesis regulation of translation**				
PRKRIR	protein-kinase, interferon-inducible double stranded RNA dependent inhibitor, repressor of (P58 repressor)	(See description above)	Hs.177574	5612
EEF1D	eukaryotic translation elongation factor 1 delta (guanine nucleotide exchange protein)	(See description above)	Hs.334798	1936
NQO1	NAD(P)H dehydrogenase, quinone 1	(See description above)	Hs.406515	1728
LIPA	lipase A, lysosomal acid, cholesterol esterase (Wolman disease)	N-linked glycosylation; hydrolase activity; lipid catabolism; lipoprotein lipase activity; lysosome; sterol esterase activity	Hs.85226	3988
LAMR1	laminin receptor 1 (ribosomal protein SA, 67 kDa)	(See description above)	Hs.181357	3921
EEF2*	eukaryotic translation elongation factor 2	translational elongation, GTP binding translation elongation factor activity, protein biosynthesis	Hs.75309	1938
EIF5A*	eukaryotic translation initiation factor 5A	regulation of translational initiation, nucleic acid binding, translation initiation factor activity, cytoplasm, viral genome replication, protein biosynthesis, translational initiation	Hs.534314	1984
**14)Cytoskeleton**				
KFC3	kinesin family member C3	ATP binding; microtubule associated complex; motor activity	Hs.23131	3801
TUBB-5	tubulin beta MGC4083	(See description above)	Hs.274398	84617
KRT5	keratin 5 (epidermolysis bullosa simplex, Dowling-Meara/Kobner/Weber-Cockayne types)	epidermal differentiation; intermediate filament; structural constituent of cytoskeleton	Hs.433845	3852
**15)Transport**				
TIP47	mannose-6-phosphate receptor binding protein 1	(See description above)	Hs.140452	10226
SRP14	signal recognition particle 14 kDa (homologous Alu RNA binding protein)	(See description above)	Hs.180394	6727
TUBB-5	tubulin beta MGC4083	(See description above)	Hs.274398	84617
FABP5	fatty acid binding protein 5 (psoriasis-associated)	(See description above)	Hs.408061	2171
**16) Cell Death**				
ZAK	sterile alpha motif and leucine zipper containing kinase AZK	(See description above)	Hs.115175	51776
MALT1	mucosa associated lymphoid tissue lymphoma translocation gene 1	(See description above)	Hs.180566	10892
LYZ	lysozyme (renal amyloidosis)	(See description above)	Hs.234734	4069

Genes in blue rectangles are up-regulated in MP stimulated by "signature IFNs" (and down-regulated in non-signature interferons). Genes in orange rectangles are up-regulated in MP stimulated by non-signature IFN (and downregulated in signature interferons). Genes belonging to more than one functional group may represent central and shared switches in pathways directly influenced by IFNs' activity. Genes in torqoise characters did not classify in any of the categories mined by DAVID software (PSMA3, NKTR, NKG7, CD63, EEF2) and were manually entered in the appropriate group on the basis of Entrez cross database annotation and Genecard ;

Signature IFNs upregulated all the genes classified in 7 out of 16 categories: 5) Cell cycle, 6) Regulation of transcription, 7) Nuclear binding, 8) Nucleobase/ nucleoside and nucleic acid metabolism, 9 Signal transducer activity (except for one gene), 14) Cytoskeleton, and 15) Transport. These results indicate that not only are signature IFNs capable of affecting simultaneously a variety of vital cellular functions in MPs but these IFNs are powerful inducer of gene transcription and key regulators of cellular responses to diverse types of stimuli.

Both the signature and non-signature IFNs upregulated MP genes in 7 categories 1) Metabolism/protein metabolism; 2) Catalytic activity/hydrolase activity 3) Cell proliferation, 4) Cell growth and maintenance 10) Immune response/defense, 11) Cell adhesion, and 13) Biosynthesis/regulation of translation. Twenty four genes were classified into the 1) Metabolism/protein metabolism, the functional category that contained the greatest number of genes. The large majority of metabolic genes belonged to vital pathways of cell survival. Activation of metabolic genes by the signature and non-signature IFNs affected distinct and yet equally important groups of genes. Seventeen metabolic genes were upregulated by signature-IFNs and these 17 genes affected major metabolic pathways such as MAP/ERK/MAPKK pathway (ZAK), glycolysis/glucose metabolism (GAPD), NADPH dehydrogenase/ oxidoreductase activity (NQO1), ubiquitin dependent protein catabolism/ proteasome endopeptidase activity (PSMA7, PSMA3), lipid metabolism (FABP5), endocytic machinery of cell trafficking (CD63) and nuclear DNA binding related to cell differentiation and proliferation (TAL1, DIS155E). Seven out of 24 metabolic genes were upregulated by non-signature-IFNs and were primarily related to enzymatic activities in protein biosynthesis (LAMR1), proteolysis and peptidolysis (MALT1), lipid catabolism(LIPA) and cytolysis (LYZ).

The functional categories 10) Immune genes and 11) Cell adhesion are of particular significance because they may be the basis of the selective immune-polarization of MPs induced by the different IFNs. In particular, immune genes such, IL-6, chemokine receptor 1 (CCR1), chemokine ligand 1 (CCL1) [50], CD2BP2 and chemokine ligand 3-like 1 (CCL3L1), were specifically expressed by the signature IFNs. CCR1 encodes a member of the beta chemokine receptor family, that binds macrophage inflammatory protein 1 alpha (MIP-1 alpha), regulated on activation normal T expressed and secreted protein (RANTES), monocyte chemoattractant protein 3 (MCP-3), and myeloid progenitor inhibitory factor-1 (MPIF-1). CCRI is a critical receptor for the recruitment of effector immune cells to the site of inflammation [10, 51, 52 Cell Migration Consortium ] CCL3L1 binds to several chemokine receptors including chemokine binding protein 2 and chemokine (C-C motif) receptor 5 (CCR5). CCR5 is a co-receptor for HIV, and binding of CCL3L1 to CCR5 inhibits HIV entry [52,53]. Thus regulation of CCL3L1 by signature-IFNs may represent a selective mechanism of viral immunity.

The activation of transcription of CCL1 and CD2BP2 in MPs by signature IFNs is an interesting finding considering that these two genes are normally activated in T cells. CCL1/ inflammatory cytokine I-309 is normally released by activated T cells during inflammation [52], and the CD2BP2 adaptor binds the CD2 cytoplasmic tail and regulates interleukin-2 production [54]. The modulation of CCL3L1, CCR1, CCL1, CD2BP2 by the signature-IFNs confirms the dominant role of these factors in the first phase of the innate immune response, inflammation, viral defense, and regulation of inflammatory cytokines such as IL-2.

Macrophage expressed gene 1 (MPEG1) [55,56] (NKG7) [57], NKTR [58], MALT-1, LYZ AND CD164 were upregulated by the non-signature group suggesting that these genes with potential cytotoxic and inflammatory functions may increase the effector function of MPs. It is important to notice that IFN-αWA and IFN-αK behaved as the non-signature IFNs regarding activation of immune genes (NKTR, CD164 and MPEG) and thus did not cluster with the signature IFNs (Figure 4). These two IFN alphas may contribute to a special type of MP polarization which may be leading to an intermediate phase of activation in between alternative and classical pathways. The non-signature IFNs also induced the expression of genes involved with cell adhesion and complement regulation: LAMR1 [59], ICAM3 [60], CD164 [61] and complement component 1 receptor subcomponent (C1R) [62] providing further evidence that IFNs belonging to the non-signature-like group may polarize MPs towards the classical pathway of monocyte activation.

Signature IFNs specifically affected a group of genes involved in eukaryotic translation and listed in category 13) biosynthesis/regulation of translation: eukaryotic translation initiation factor 5A (EIF5A) [63], eukaryotic translation elongation factor 1 delta (EEF1D) [64], and eukaryotic translation elongation factor 2 (EEF2) [65]. This supports the notion that the powerful stimulus of activation induced by the signature IFNs on immune cells is a global type of regulation directly affecting target molecules at the levels of both transcription and translation.

Genes upregulated by signature IFNs and belonging to more than one functional group were particularly interesting because they could represent central and shared molecular and cellular switches in pathways not previously reported to be influenced by the signature-IFNs' activity. The ZAK (sterile alpha motif and leucine zipper containing kinase AZK) gene appeared to be a very dominant signature IFNs gene since it was shared by 9 out of 16 functional groups analyzed. ZAK encodes a mixed lineage kinase with a leucine zipper and a sterile alpha motif. The expression of ZAK in mammalian cells may lead to the activation of the JNK/SAPK (MAP kinase family) pathway as well as the activation of the transcription factor, NF-kappaB. Furthermore over-expression of ZAK leads to apoptosis in tumor cell lines [66].

### Phylogenic analysis

**Figure 5:**
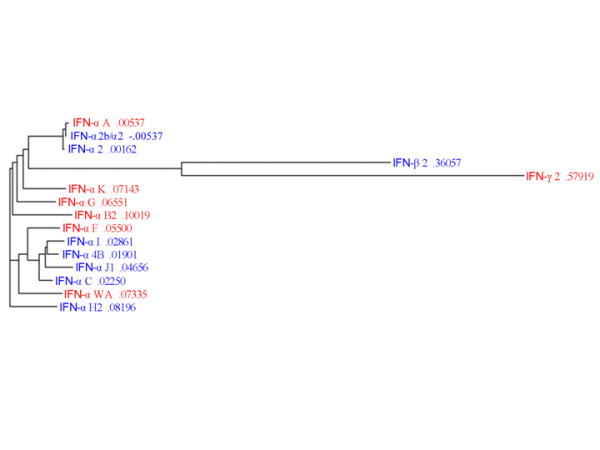
**Phylogenic comparison of IFNs**. A multiple alignment cluster of the amino acid sequences of the 13 αIFNs, βIFN, and γIFN was preformed with Cluster W. The signature IFNs are shown in blue and the non-signature IFNs are shown in red.

**Figure 6:**
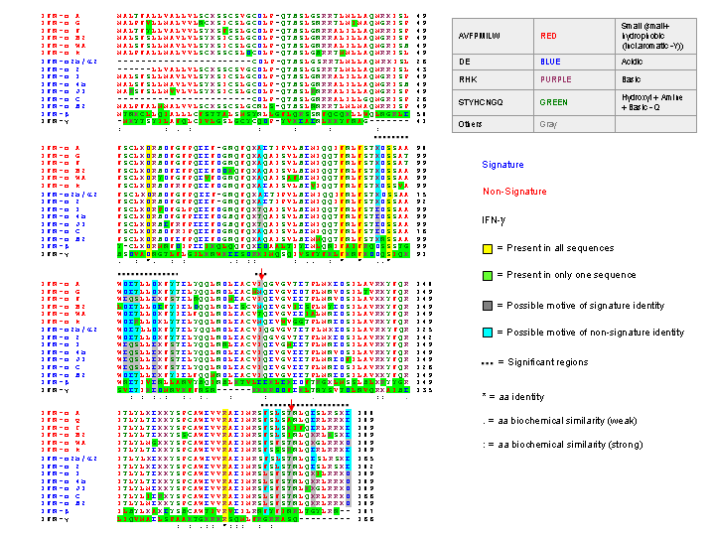
**Comparison of the amino acid sequences of IFN-β and the 13 α-IFNs studied**. The signature IFNs are shown in blue and the non-signature IFNs are shown in red. Amino acids that were more likely to be present in signature IFNs are shown in gray and those more likely to be present in non-signature IFNs are shown in torqoise. Amino acids present in IFN-β, y and all 13 αIFNs are shown in yellow.

To determine whether a correlation existed between closeness of the primary amino acid sequence of type I IFNs and functional similarities we compared the sequence relationship among the 13 αIFNs and IFN-β. The phylogenic analysis did not predict the differentiation between signature and non-signature IFNs and, in fact, the signature IFNs included proteins phylogenically quite unrelated **(**IFN-αWA, IFN-αF and IFN-αB2 Figure 5). Therefore, more limited domains of the structure of each IFN may be responsible for their different functional properties. Comparison of the primary amino acid sequences of IFN-β and the 13 αIFNs identified several similarities (Figure 6). Although there was no exclusive sequence variation that could distinguish signature from non-signature IFNs, some amino acids were more likely present in one or the other group. Three regions appeared to be most significant in distinguishing the subgroups of the protein and included: residues 93 through 109, residues 174 through 188-9, and at residue 123. The differences were greatest at residues 123 and 179 where signature IFNs (except IFN-β) had the same amino acid (I at 124 and T at 179, red arrows, figure 6), but these amino acids were present in only 2 of 6 non-signature αIFNs.

## Discussion

Interferons are important cytokines with antiviral, anti-proliferative, and immune modulatory functions [67]. The IFNs are divided into two classes, type I and type II. The type I IFNs include the α, β, τ and ωIFNs, while IFN-γ is the only type II IFN. It has generally been believed that the various type I IFNs have markedly different biological functions and the immune modulatory effects of type I IFNs vary widely among individual IFNs. IFNs exert many of their effects by modulating MP functions [1,68]. The stimulation of MPs by cytokines, including IFNs, results in the activation of several different pathways; some of which are common to different stimuli and some that are unique for each stimuli. This study shows that most type I IFNs share some MP activation pathways and functional differences among IFNs are due to additional diversification of their function through other pathways of activation. In particular, the effects of seven IFN-α and IFN-β on LPS-stimulated MPs were remarkably similar. These similarities were not apparent after 4 hours of stimulation and required a longer process of down-stream activation that could be identified in our study at 9 hours.

Since we only studied one subject, the transcriptional profile presented in this study may not represent the plethora of biological effects that IFN can induce under *in vitro or in vivo *conditions because secondary autocrine (in different donors) and paracrine modulation through the cytokine network following a primary stimulation may introduce diverse on and off switches that could override the original signal. Nevertheless, this is an informative analysis on the effects of IFN subtypes on the late stages of LPS stimulation and, therefore, maybe most informative on the way mononuclear phagocytes are polarized by IFNs during their activation and or maturation.

A group of 42 signature genes was upregulated by all the tested IFNs. Furthermore, all 42 signature genes have previously been found to be associated with IFNs (Table 1). The analysis of a group of 83 genes that were differentially expressed among the signature and non-signature IFNs found that the signature IFNs affected a variety of cell functions not only by being potent inducers of gene transcription and translation but by directly and indirectly affecting crucial regulatory cell cycle and metabolic pathways. In addition, signature IFNs were found to be particularly involved in the first phase of the innate immune response, inflammation, and viral defense. In contrast, the non-signature IFNs gene profiling suggested that these immune-modulators can trigger cytotoxic responses and likely increase the effector functions of MPs or classical pathways of monocyte activation.

Type I IFNs play an important role in autoimmune disorders. Serum levels of IFN-α are increased in some patients with the autoimmune SLE. The gene expression profiles of peripheral blood MPs is also abnormal in patients with SLE. Analysis of peripheral blood MPs from patients with SLE has found that IFN-inducible genes are upregulated in almost all patients with SLE [2,3,67] and the genes upregulated in SLE patients were similar to those upregulated in peripheral blood MPs stimulated in vitro with IFN-α [1-3,67]. As a result the IFN-inducible genes that are upregulated in SLE have been called IFN signature genes. Many of the IFN-inducible genes up regulated in SLE patients are the same genes we found were upregulated by the treatment of LPS-stimulated MPs with the eight signature IFNs. The genes upregulated both in MPs from patients with SLE and by LPS-stimulated genes treated with the signature IFNs included OAS1, OAS2, LY6E, MX1, CIG5, and hepatitis C-associated microtubular aggregation protein (44 kD) [2,3,67].

Hilkens et al studied the effects of three IFNs, the signature IFNs, IFN-α1, IFN-α2, and the non-signature IFN, IFN-αF on the expression of 150 IFN stimulated genes by dendritic cells derived from peripheral blood mononuclear cells and found that the qualitative effects of the three IFNs were similar but there were quantitative differences [69]. The IFN stimulated genes were more highly induced by IFN-α2 and IFN-αF than IFN-α1, but these differences were overcome by increasing the concentration of IFN-αI. There was, however, one exception. The expression of IP-10 was induced by IFN-α2 and IFN-αF, but not by IFN-αI. This supports our finding that the differences in transcriptional profiles of MPs treated by different type I IFNs are due to distinct effects of each of the IFNs rather than differences in the activation of IFN-specific genes.

Although increased serum levels of IFN-α and IFN-ω have been found in some patients with SLE [67], the proportion of patients with detectable IFN in serum is much smaller than the proportion of SLE patients with peripheral blood MPs that display evidence of IFN-stimulation by gene expression profiling [1]. However, since the IFN genes upregulated in MPs from SLE patients were similar to those upregulated by MPs stimulated with IFN-α, most studies have measured IFN-α serum levels in patients with SLE. Our study suggests other type I IFNs in addition to IFN-α could be responsible of the abnormal MP gene expression profiles in patients with SLE. It may be that IFNs other than IFN-α are increased in SLE patients but the ELISA assays used to measure IFN-α may not detect other type I IFNs that could result in a similar MP gene expression profile. One commonly used IFN-α ELISA kit recognizes IFN-αA, IFN-α2, IFN-αA/D, IFN-αD, IFN-αK, and IFN-α4b, but not IFN-β or IFN-γ (BioSource International USA, Inc., Camarillo, CA) another kit recognizes IFN-αA, α2, αA/D, αB2, αC, αD, αG, αH, αI, αJ, αK, αWA, and α4b (PBL laboratories multi species IFN α elisa kit).

Type I IFNs belong to a family of homologous helical cytokines. The various αIFNs have approximately 80% amino acid sequence homology and the α and βIFNs have approximately 30% sequence homology [70]. The α and βIFNs, however, all have a similar three-dimensional structure. They are made up of nearly parallel bundles of five α helices. In addition to the shared tertiary structure, the α and βIFNs signal cells through the same receptor complex, the common IFN-α/β receptor (IFNAR). This receptor is made up of two subunits IFNAR1 and IFNAR2. IFNAR2 plays a greater role in binding type I IFNs than IFNAR1. Although IFNAR2 is the major IFN binding component of the receptor, the formation of the tertiary complex of IFNAR2 and IFN with IFNAR1 increases the affinity of IFN to IFNAR2 up to 20-fold.

The binding of IFN-α2 to IFNAR1 and IFNAR2 has been extensively investigated [64-68]. IFNAR2 binds to the A helix of IFN-α2 (residues 12–15), the AB loop (residues 26–35), and the E helix (residues 144–153) [71]. The opposite face of IFN-α2, the B and C helices, is involved with interactions with IFNAR1 [70]. Modeling of the interactions of IFNAR2 and IFN-α2 based on multidimensional NMR analysis of these molecules found that IFN-α2 residues R33, D35 and R149 interact with a hydrophilic strip of matching charges in IFNAR2. Other important IFN-α2 residues involved with the binding to IFNAR2 are M16 and A19 of the A helix, L26 and L30 of the AB loop, and A145 and M148 of the E helix. Mutagenesis analysis of IFN-α2 found six residues important in IFNAR2 binding: L30, R33, R144, A145, and M148. The residues we found that differ between the signature and non-signature IFNs are in areas that are not critical to the binding of IFN-α2 to IFNAR2. Our findings suggest that changes in IFN-α2 residues in the carboxy end (residues 174, 177, 178-9, 183 and 189), the D helix (residue 124) and the C helix (residues 94, 101, 102, 103, 106, and 109) may affect the function of α and βIFNs and are be responsible for the functional similarities among the signature IFNs. We speculate that these changes may affect the tertiary structure of type I IFNs and there binding by IFNAR1.

## Conclusion

MPs demonstrate a biphasic response to cytokine stimulation and 9 hours after IFN treatment LPS-stimulated MPs had a striking gene expression profile. When treated with eight type I IFNs the same group of genes were strongly upregulated. IFNs play a critical role in host defense and the redundant function of these signature IFNs may help insure that there are multiple pathways of activation of potent host antiviral and antitumor responses. Since several different IFNs have similar effects, when clinical or molecular evidence suggest that serum IFN levels are increased, assays that detect a wide range of type I IFNs should be used to measure soluble IFN levels.

## List of Abbreviations

Mp = mononuclear macrophage

PBMC = peripheral blood mononuclear cells

IFN = interferon

CM = Complete medium

OM = OPTI-MEM

LPS = lipopolysaccharide

TNF = tumor necrosis factor
